# Targeting NAD Homeostasis: Compartmentalization, Quantification, and Modulation

**DOI:** 10.3390/metabo16050338

**Published:** 2026-05-18

**Authors:** Marta Nobile, Veronica Fontanini, Simone Serrao, Johannes Burtscher, Francesca Re, Giuseppe Paglia

**Affiliations:** 1 School of Medicine and Surgery, University of Milano-Bicocca, Via Raoul Follereau, 3, 20854 Vedano al Lambro, MB, Italy; m.nobile13@campus.unimib.it (M.N.); veronica.fontanini@unimib.it (V.F.); simone.serrao@unimib.it (S.S.); francesca.re1@unimib.it (F.R.); 2 Department of Psychiatry, Psychotherapy, Psychosomatics and Medical Psychology, University Clinic for Psychiatry II, Anichstraße 35, 6020 Innsbruck, Austria; johannes.burtscher@i-med.ac.at; 3 Fondazione IRCCS San Gerardo dei Tintori, Via Pergolesi 33, 20900 Monza, MB, Italy

**Keywords:** NAD^+^/NADH, NAD metabolism, subcellular compartmentalization, NAD quantification, NAD modulation, metabolic regulation

## Abstract

**Highlights:**

• NAD^+^/NADH regulates cellular redox balance, energy metabolism, and key signaling processes.
• Highly compartmentalized NAD pools exist across organelles with distinct con-centrations and redox states.
• NAD quantification is challenged by methodological variability, rapid intercon-version, and dynamic cellular states.
• NAD^+^-boosting strategies show therapeutic promise but face significant transla-tional and compartmentalization challenges.

**Abstract:**

Nicotinamide adenine dinucleotide (NAD^+^) and its reduced form, NADH, are essential coenzymes that play central roles in cellular redox homeostasis, energy metabolism, DNA repair, and signaling. Cellular NAD^+^ levels are maintained by a dynamic balance between the de novo Preiss–Handler, and salvage synthesis pathways, and consumption by enzymes like sirtuins, PARPs, and CD38. Among these, the nicotinamide Phosphoribosyltransferase (NAMPT)-driven salvage pathway represents the predominant route of NAD+ synthesis. The specific regulation of NAD (NAD^+^ and NADH) levels across distinct subcellular compartments has emerged as a critical determinant of cellular function but it remains poorly understood. Dysregulation of NAD metabolism is a hallmark of aging and various pathologies, including cancer, neurodegenerative disorders, and metabolic diseases, making strategies to modulate NAD levels a promising therapeutic frontier. This review provides the first integrated overview of NAD concentrations across cellular compartments (cytosol, mitochondria, nucleus, endoplasmic reticulum, Golgi, peroxisomes, and the extracellular space) together with measurement and modulation strategies. We summarize current knowledge on NAD distribution within organelles, address key challenges in accurate quantification, and highlight established and emerging approaches for both global and compartment-specific analysis. Finally, we discuss therapeutic strategies, from NAD^+^ precursor supplementation to enzyme modulators and gene therapy, highlighting both their translational potential and current limitations in treating diverse diseases and prolonging life and health span.

## 1. Introduction

Nicotinamide adenine dinucleotide (NAD) in its oxidized form (NAD^+^) and its reduced form (NADH) are essential coenzymes involved in virtually all aspects of cellular biology. NAD^+^ and NADH are central to cellular energy metabolism: NAD^+^ accepts electrons in catabolic pathways like glycolysis and the Tricarboxylic Acid Cycle (TCA) cycle, forming NADH, which then donates electrons to the electron transport chain for ATP generation [1]. Maintaining a physiological NAD^+^/NADH ratio is essential for efficient mitochondrial respiration and overall cellular energy homeostasis.

Beyond their crucial roles as regulators of cellular redox homeostasis and metabolism, NAD^+^ and NADH participate in DNA repair, cell cycle progression, gene expression, cell signaling, etc. [1].

The cellular NAD^+^ pool is tightly regulated through a dynamic balance between synthesis and degradation [2]. NAD^+^ can be synthesized via three main pathways: the de novo pathway starting from tryptophan, the Preiss–Handler pathway utilizing nicotinic acid (NA) [3], and the predominant salvage pathway, which recycles nicotinamide (NAM) and nicotinamide riboside (NR) [4]. The salvage pathway, primarily catalyzed by nicotinamide phosphoribosyltransferase (NAMPT), is particularly important in mammals for maintaining cellular NAD^+^ levels [5]. Approximately 85% of total NAD^+^ is produced through this pathway [6].

**Figure 1:**
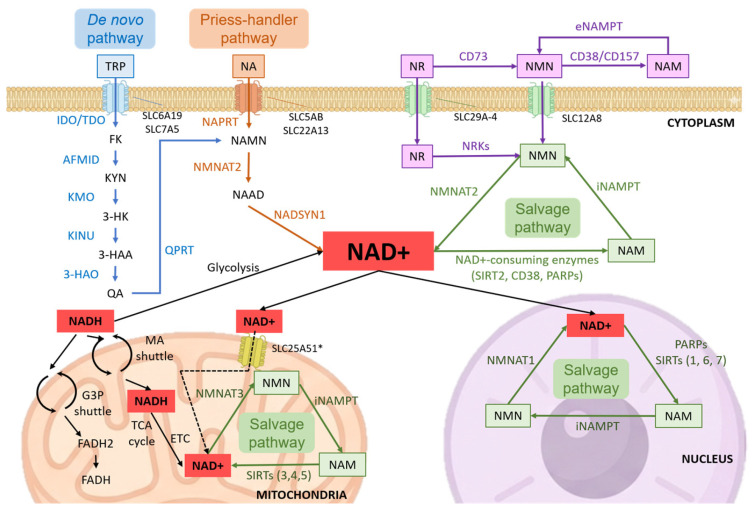
Integrated overview of NAD^+^ metabolism and compartmentalization. Schematic representation of NAD^+^ biosynthesis through the de novo, Preiss–Handler, and salvage pathways. The diagram illustrates the compartmentalized distribution of NAD^+^ across cytosol, mitochondria, and nucleus, highlighting the organization of distinct subcellular NAD^+^ pools and their functional interplay with NAD^+^-consuming enzymes. Extracellular NAD^+^ metabolism and precursor interconversion pathways are also depicted [14,27]. Abbreviations: NAD^+^, nicotinamide adenine dinucleotide; NADH, reduced NAD^+^; NA, nicotinic acid; NAM, nicotinamide; NMN, nicotinamide mononucleotide; NAMN, nicotinic acid mononucleotide; NAAD, nicotinic acid adenine dinucleotide; NR, nicotinamide riboside; NAMPT, nicotinamide phosphoribosyltransferase; NMNAT1–3, nicotinamide mononucleotide adenylyltransferases; NAPRT, nicotinic acid phosphoribosyltransferase; NADSYN1, NAD synthetase 1; NRKs, nicotinamide riboside kinases; CD38/CD157, NAD^+^ glycohydrolases; CD73, ecto-5′-nucleotidase; SIRT(s), sirtuins; PARP(s), poly(ADP-ribose) polymerases; SLC25A51, mitochondrial NAD^+^ transporter; TCA cycle, tricarboxylic acid cycle; ETC, electron transport chain. * SLC25A51 has been recently identified as a mitochondrial NAD^+^ transporter; however, the relative contribution of NAD^+^ import versus local synthesis remains under investigation.

Importantly, two mechanistically distinct processes regulate NAD^+^ turnover: redox cycling, in which NAD^+^ is reversibly reduced to NADH by metabolic enzymes, and enzymatic consumption, in which NAD^+^ is cleaved by NAD^+^-consuming enzymes (e.g., sirtuins, PARPs, CD38) to produce nicotinamide (NAM), which re-enters the salvage pathway. This distinction is critical for accurately interpreting NAD dynamics and fluxes (Figure 1) [7,8].

Dysregulation of the NAD^+^/NADH ratio has been implicated in the pathogenesis and progression of numerous age-related diseases [5]. A decline in NAD^+^ levels is one of the most consistent and evolutionarily conserved hallmarks of aging across species, from yeast to humans, and is observed in virtually all tissues with advancing age [9,10,11]. This age-associated NAD^+^ depletion—typically ranging from 30 to 70% in old versus young organisms—contributes directly to multiple hallmarks of aging, including mitochondrial dysfunction, impaired DNA repair, loss of proteostasis, deregulated nutrient sensing, chronic inflammation (inflammaging), and cellular senescence [10,11,12]. The decline is driven by multiple interconnected mechanisms, including reduced efficiency of the NAMPT-mediated salvage pathway, increased activity of NAD^+^-consuming enzymes (notably CD38 and PARPs), and alterations in cellular bioenergetics and redox balance. These changes are tightly linked to broader metabolic rewiring, including impaired glycolysis, mitochondrial dysfunction, and altered lipid metabolism, highlighting the central role of NAD^+^ in cellular bioenergetics [13,14,15]. In cancer, by contrast, altered metabolic demands lead to dramatically increased NAD^+^ turnover to sustain rapid proliferation and biosynthetic needs [16]. In neurodegenerative disorders such as Alzheimer’s and Parkinson’s disease, compromised NAD^+^ availability exacerbates neuronal vulnerability, mitochondrial failure, and impaired proteostasis [17]. Similarly, disrupted NAD^+^ homeostasis promotes oxidative stress and insulin resistance in metabolic disorders, including obesity [18], type 2 diabetes [19], and cardiovascular diseases [20]. Genetic or pharmacological restoration of NAD^+^ levels in aged and progeroid models improves mitochondrial function, stem cell activity, neurovascular coupling, physical endurance, and both healthspan and lifespan, establishing NAD^+^ replenishment as one of the most promising interventions against age-related functional decline [20,21,22].

Accordingly, modulating NAD^+^ levels and the activity of NAD-metabolizing enzymes has emerged as a promising therapeutic approach to a variety of disorders. However, despite extensive research, a key limitation in the field is the incomplete integration of three critical aspects: (i) subcellular compartmentalization of NAD pools, (ii) methodological challenges in accurately quantifying NAD species, and (iii) the impact of these factors on the efficacy and specificity of therapeutic approaches.

Cellular compartmentalization represents a fundamental cellular strategy to protect and regulate distinct NAD pools. Simply increasing total intracellular NAD^+^ levels, therefore, may not affect all subcellular pools equally or as desired.

Importantly, emerging evidence suggests that the efficacy of NAD-targeting interventions is highly dependent on the distribution and accessibility of NAD within specific subcellular compartments, which can differentially regulate metabolic and signaling pathways.

For instance, mitochondria play a crucial role in maintaining cellular NAD levels and buffering against shortages in other cellular compartments. Although cells can generally tolerate a drop in overall NAD^+^, a decline in the mitochondrial NAD^+^ pool is especially detrimental [23]. Even under stress conditions, mitochondrial NAD^+^ remains stable longer than NAD^+^ in the nucleus or cytoplasm. This indicates a built-in protective system where mitochondrial NAD^+^ is used only as a last resort [24,25,26].

Therefore, effective strategies for controlled NAD modulation require precise quantitative mapping of NAD levels within each compartment. Reliable and accurate measurement of NAD concentrations is equally crucial as the foundation for assessing metabolic dynamics and designing targeted interventions.

In this context, this review aims to address this critical gap by providing an integrated framework that connects NAD compartmentalization, quantification methodologies, and therapeutic modulation strategies. It provides a topical overview of NAD concentrations across different cellular compartments, presents a comprehensive assessment of the technologies available for their measurement, and examines existing approaches for the modulation of NAD metabolism. Together, these elements contribute to establishing a reference point that can guide both fundamental research and the development of therapeutic strategies.

## 2. Intracellular and Organelle-Specific NAD^+^ and NADH Concentrations

**Figure 2:**
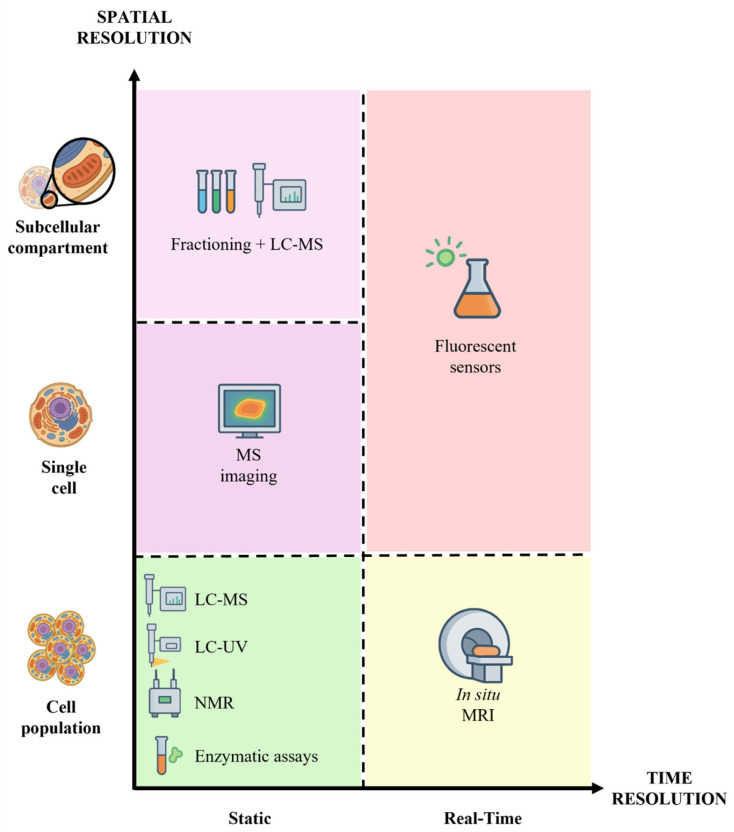
Comparison of NAD^+^ quantification methods based on spatial and temporal resolution: in situ Magnetic Resonance Imaging (MRI), Liquid Chromatography tandem Mass Spectrometry (LC-MS), LC-UV, Nuclear Magnetic Resonance (NMR), enzymatic assays, Mass Spectrometry Imaging (MSI), fluorescent sensors, fractioning, and LC-MS [29].

Accurate measurement of NAD, both at the cell/tissue and organelle level, is essential for understanding cellular redox homeostasis and metabolism, and roles in physiopathology. This is challenging due to low abundance, rapid interconversion, and compartmentalization [28]. No single method is currently capable of capturing the full complexity of NAD metabolism; instead, each approach involves inherent trade-offs between temporal resolution, spatial localization, sensitivity, and quantitative accuracy. Importantly, these methodological differences can lead to substantial variability in reported NAD concentrations, which must be carefully considered when comparing studies (Figure 2, Table 1 and Table 2) [29].

The concentration of NAD varies significantly not only between mammalian species but also among cell types within the same species [30]. Moreover, NAD levels in cell lines are often reported relative to cell number (e.g., moles per million cells) rather than cell volume, complicating comparisons with other concentration units [31]. In addition, analytical platforms (e.g., LC-MS, enzymatic assays, biosensors) exhibit substantial differences in specificity, sensitivity, dynamic range, and sample preparation requirements, introducing systematic biases across studies. Sample preparation steps, particularly quenching and extraction, can induce artifactual interconversion between NAD^+^ and NADH, thereby distorting the measured redox state [32,33]. Furthermore, a critical but often overlooked factor is whether measurements reflect total or free NAD pools [28,34]. A significant portion of cellular NAD is protein-bound and tightly associated with enzymatic complexes, especially in mitochondria, while only the free pool is metabolically active [33]. Conventional extraction-based methods predominantly quantify total NAD, potentially overestimating the metabolically available fraction, whereas selective biochemical or imaging-based methods can differentiate between pools, aiding interpretation of redox balance [34,35] (Table 1 and Table 2).

Additionally, disruption of subcellular compartmentalization during sample processing represents a major limitation, as it obscures the true intracellular distribution of NAD species and may lead to misinterpretation of compartment-specific dynamics. Beyond these methodological considerations, NAD levels are highly dynamic and depend on cellular metabolic states, nutritional status, age, and environmental conditions [36].

Consequently, measuring the NAD^+^/NADH ratio is often preferred because it provides a specific readout of the redox state and is generally easier to interpret. From a methodological perspective, ratio-based measurements can partially mitigate absolute quantification biases. However, they remain sensitive to experimental artifacts such as differential degradation or interconversion of NAD species [37].

To study NAD metabolism, many experimental models exist, in vivo, ex vivo, or in vitro [28], but a trade-off is necessary to obtain good results on temporal resolution, tracking changes over time, and spatial resolution, which allows NAD localization within tissues or subcellular compartments [38]. Each technique, therefore, provides a partial and context-dependent view of NAD metabolism, reinforcing the need for integrative approaches that combine complementary methodologies. In Table 1 and Table 2 and Figure 1, the wide range of technologies used for NAD measurement, considering their advantages and limitations, is summarized.

In mammalian cells, total NAD amounts to between approximately 200 and 800 µM [26,39,40,41]. However, reported values vary widely depending on the measurement method and experimental conditions. For example, LC-MS-based studies and biosensor-based approaches can yield substantially different estimates, reflecting methodological biases rather than true biological differences. Notably, substantial discrepancies exist across studies reporting NAD^+^ concentrations, reflecting methodological rather than purely biological differences. For instance, Yang et al. reported total cellular NAD^+^ levels of approximately 365 ± 30 µM in HEK293 cells, with mitochondrial concentrations around 245 µM, whereas biosensor-based approaches, such as those used by Cambronne et al., tend to report lower free NAD^+^ concentrations due to selective detection of metabolically active pools [26,31]. Similarly, LC–MS-based approaches generally yield higher absolute NAD^+^ concentrations compared to genetically encoded biosensors, which primarily capture relative changes and free NAD^+^/NADH ratios in live cells. These differences highlight the critical distinction between total and free NAD^+^ measurements, as well as the impact of extraction procedures and subcellular resolution on quantitative outcomes. Therefore, direct comparison between studies requires careful consideration of both the analytical method and the specific NAD pool being measured, as these discrepancies complicate the establishment of standardized reference values for intracellular and compartment-specific NAD^+^ concentrations. Importantly, only an estimated 10–25% of this pool exists as free NAD, the metabolically active fraction directly available for redox reactions, enzymatic consumption by sirtuins, PARPs, and CD38, and signaling. In contrast, the majority of NAD is protein-bound, particularly in dehydrogenases, and is less accessible for rapid metabolic regulation. This distinction is critical because most conventional extraction-based methods do not discriminate between these pools, complicating the interpretation of functional NAD^+^/NADH ratios [41,42]. This balance between free and bound NAD is highly dynamic and depends on different conditions, such as hypoxia or age [43,44].

Approximately 10% of the total NAD pool is phosphorylated by NAD kinases to generate NADP^+^ and NADPH [4,41,45]. While the NAD^+^/NADH couple is mainly dedicated to catabolic reactions and mitochondrial energy production, the NADP^+^/NADPH redox pair primarily supports anabolic processes such as fatty acid, cholesterol, and nucleotide biosynthesis. Moreover, NADPH plays a central role in cellular antioxidant defense by providing reducing power for glutathione reductase and thioredoxin systems, and it serves as an electron donor for NADPH oxidases during immune responses [45,46]. Significantly, the ratios NAD^+^/NADH and NADP^+^/NADPH represent key indicators of cellular redox state and metabolic flux. Alterations in these ratios reflect shifts between oxidative and reductive metabolism and have been proposed as potential biomarkers of metabolic dysfunction and disease progression, including conditions characterized by mitochondrial impairment, oxidative stress, or altered biosynthetic demand [45,47].

Importantly, the NADP(H) pool is largely insulated from the NAD(H) pool due to the substrate specificity of most dehydrogenases, allowing independent regulation of catabolic and anabolic redox states. Collectively, NADPH-dependent pathways are particularly important during cellular stress, when biosynthetic, antioxidant, and immune demands increase [46,48].

**Table 1:** Whole-cell (intracellular) NAD^+^ and NADH quantification.

I. Whole-Cell (Intracellular) NAD^+^ and NADH Quantification				
Method	Principle	Advantages	Challenges	LOD, LOQ, Dynamic Range
Enzymatic Cycling Assays (colorimetric/fluorometric/luminescent) [33,49,50]	Based on coupled enzyme reactions that cyclically convert NAD^+^/NADH into a detectable product (absorbance or fluorescence signal). Samples: cultured cells, tissue homogenates, plasma (kit-dependent).	-High sensitivity and throughput. -Inexpensive and easy to perform. -Suitable for large sample numbers. -Moderate specificity for free NAD^+^, depending on acid/base extraction.	-Low chemical specificity compared to chromatographic methods. -Requires separate acid/base extraction for NAD^+^ and NADH. -Prone to variability due to incomplete degradation or instability.	LOD: 1 nM (25 fmol/25 µL) (e.g., NAD/NADH-Glo™) Dynamic range: 1–500 nM
High-Performance Liquid Chromatography (HPLC-UV) [26,51]	Chromatographic separation of NAD^+^ and NADH based on UV absorbance (260 nm for NAD^+^, 340 nm for NADH). Samples: cultured cells, tissue extracts (liver, muscle, brain), yeast.	-Quantitative and well-established method. -Can distinguish oxidized/reduced species. -High specificity for total NAD^+^.	-Limited sensitivity and selectivity in complex samples -Potential interference from other nucleotides. -No subcellular resolution.	Not reported
High-resolution liquid chromatography coupled with tandem mass spectrometry (LC-MS/MS) [40,52,53,54]	High-resolution liquid chromatography coupled with tandem mass spectrometry for precise identification and quantification of NAD^+^, NADH, and related metabolites. Samples: cultured cells, tissue (liver, brain, muscle, adipose), plasma, whole blood, urine.	-Gold standard for absolute quantification. -Very high sensitivity and chemical specificity for total NAD^+^. -Free NAD^+^ quantification limited by extraction artifacts and compartment disruption.	-Requires rapid cold acidic extraction to prevent interconversion. -Poor chromatographic retention of polar metabolites. -Risk of in-source fragmentation.	LOQ: 1 nM (in tissue extracts) Dynamic Range: 1 nM–10 µM
Mass Spectrometry Imaging (MSI) [55,56]	In situ detection of NAD^+^ and NADH in tissue sections using mass spectrometry (e.g., MALDI- or DESI-MSI), which maps metabolites based on their mass-to-charge ratio while preserving spatial distribution. Samples: fresh-frozen tissue sections (brain, liver, tumor), cryosections (10–20 µm).	-Enables label-free, spatially resolved mapping of NAD^+^/NADH in intact tissues and cells. -Preserves tissue architecture and reveals regional redox heterogeneity. -High chemical specificity for total NAD^+^ and NADH compared to optical methods. -Provides spatial context beyond bulk LC-MS analyses.	-Semi-quantitative; limited sensitivity for low-abundance, labile NAD species. -Ionization inefficiency and signal suppression hinder absolute quantification. -Requires complex sample preparation and matrix optimization. -Difficult to distinguish free from protein-bound NAD^+^.	Not reported
In vivo spectroscopy Nuclear magnetic resonance (NMR)/Magnetic resonance imaging (MRI) [57,58,59]	Nuclear Magnetic Resonance (NMR) Spectroscopy Detection of NAD^+^ and NADH through differences in magnetic resonance signals of hydrogen and phosphorus nuclei. Can be performed in vitro on cell extracts or in vivo in tissues. Samples: brain in vivo (human, rat), tissue extracts in vitro, limited to accessible regions at high field.	-Non-invasive, real-time tracking in intact tissues (e.g., brain); ^1^H and ^31^P distinguish NAD^+^ and NADH. -High chemical specificity for NAD^+^ and NADH. -Non-destructive and label-free. -Applicable in vivo for metabolic studies.	Qualitative/semi-quantitative; lacks subcellular resolution; requires high magnetic field. -Low sensitivity compared to MS-based techniques. -Requires high metabolite concentrations and long acquisition times. -Limited spatial resolution at the subcellular level. -Cannot reliably separate free NAD^+^ from protein-bound NAD^+^.	Physiological concentration: 0.324 ± 0.050 mM (Practical LOD ≈ 0.1–0.3 mM)

**Table 2:** Organelle-Specific NAD^+^ and NADH Quantification.

II. Organelle-Specific NAD Quantification				
Method	Principle	Advantages	Challenges	LOD, LOQ, Dynamic Range
Subcellular Fractionation/Immunoaffinity Organelle Isolation + Biochemical Assays or LC–MS/MS [32,33,60,61,62]	Physical or antibody-based separation of organelles from cell homogenates, followed by metabolite extraction and quantification of NAD^+^/NADH assays or LC-MS/MS. Samples: cultured cells, tissue (liver, heart, brain), isolated mitochondria, nuclei, and peroxisomes.	-Enables compartment-specific estimation of NAD^+^/NADH. -Immuno-isolation offers higher purity and faster processing, minimizing NAD degradation. -Compatible with metabolomic and isotope-tracing analyses. -Good specificity for total NAD^+^, depending on fraction purity and analytical method.	-Cross-contamination between fractions can significantly affect results. -NAD^+^/NADH are labile: ultra-fast and cold extraction is required. -Difficult to discriminate free NAD^+^ from protein-bound pools.	Depends on the downstream method
Genetically Encoded Fluorescent Biosensors (e.g., Peredox, SoNar, mito-Peredox) [5,63,64,65,66]	Fusion proteins combining an NAD^+^/NADH-binding domain with a fluorescent protein (e.g., cpVenus or cpYFP), whose emission intensity or spectral ratio changes upon NAD^+^+/NADH binding. Samples: live mammalian cells (cytosol, mitochondria, nucleus), brain slices, in vivo in zebrafish/mouse.	-Enables in situ and real-time monitoring. -Organelle targeting via localization sequences (e.g., mitochondrial, nuclear). -High specificity for free NAD^+^ or NAD^+^/NADH ratio.	-Provide relative rather than absolute concentrations. -Sensitive to pH, temperature, and O_2_ levels. -Sensor expression can perturb local NAD^+^ metabolism.	Ratio-based detection No classical LOD/LOQ
Fluorescent Probes (Small Molecules) [67,68]	Chemically synthesized fluorescent probes that react or bind to NAD(P)^+^/NAD(P)H, producing changes in fluorescence intensity, wavelength, or ratio (detected via PET, ICT, or spirolactone mechanisms). Samples: live cultured cells (HeLa, HEK, HepG2, A549, etc.), tumor tissues, paraffin-embedded sections.	-High sensitivity and selectivity for NADH or NADPH; some probes can differentiate NAD^+^/NADH. -Non-invasive and suitable for live-cell imaging. -Broad spectral range, including NIR options.	-Possible interference from other redox-active metabolites. -Complex calibration; challenging to obtain absolute concentrations. -Limited specificity for free NAD^+^ compared to NAD(P)H.	LOD: 0.36 nM (for NADH, best probe)

Importantly, methodological differences reported in Table 1 and Table 2, including sensitivity, detection limits, and sample preparation requirements, contribute significantly to variability in reported NAD concentrations across studies.

### 2.1. Organelle-Specific Concentrations and Ratios

**Figure 3:**
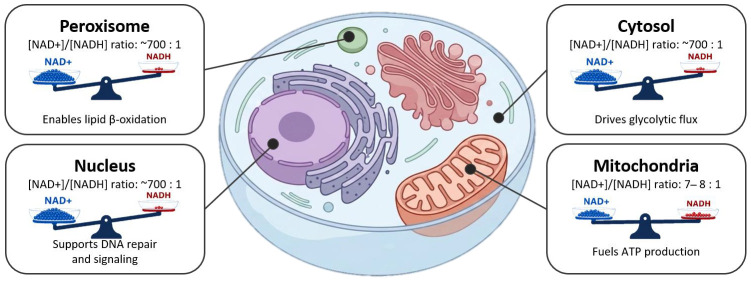
Subcellular compartmentalization of NAD^+^/NADH redox balance shapes metabolic specialization. High NAD^+^/NADH ratios in the cytosol, nucleus, and peroxisomes support glycolysis, DNA repair, and β-oxidation, whereas a lower ratio in mitochondria favors ATP production through oxidative metabolism (also see Table 3).

NAD levels vary across organelles [31,41,69] (Figure 2). This compartmentalization allows each organelle to maintain distinct NAD^+^/NADH ratios supporting specific metabolic and signaling functions. Recently, organelle-targeted biosensors and metabolomics workflows enabled the revelation of NAD distributions across [31,42,70]. Estimations of quantitative NAD metabolism parameters for individual organelles are presented in this section (Figure 3).

#### 2.1.1. Cytosol

In mammalian cells, the free cytosolic concentration is generally estimated to be around 10–100 µM in COS7 cells (Cercopithecus aethiops Renal Cells) [71] and ~100 µM in HEK293T (clonal Human Embryonic Kidney) cells [31] but can reach higher levels in other cell types, such as ~260 µM in A375 human melanoma cells [72]. Considering only free amounts, the NAD^+^/NADH ratio in the cytoplasm of healthy mammalian tissues is generally very high, often around 700:1, a state that strongly favors glycolysis, lactate dehydrogenase activity, and other dehydrogenase-catalyzed intermediates [73].

#### 2.1.2. Mitochondria

Mitochondria are major sites of NAD^+^ reduction, where metabolic enzymes of the TCA cycle reduce NAD^+^ to NADH, which is then re-oxidized by the electron transport chain to support ATP production. A significant portion of the total cellular NAD^+^ pool, estimated to be between 40% and 70%, resides within the mitochondria, although this proportion varies among cell types [31], e.g., ~70% in cardiac myocytes [73], 50% in neurons, and 30–40% in hepatocytes [74]. Importantly, the mitochondrial NAD pool is relatively distinct from the cytosolic pool, since neither NAD^+^ nor NADH can freely diffuse across the inner mitochondrial membrane [14]. While mitochondrial NAD^+^ was long thought to depend primarily on local biosynthesis through the de novo and salvage pathways, the recent identification of SLC25A51 (MCART1) demonstrated that cytosolic NAD^+^ can also be directly imported into mitochondria. However, the relative contribution of NAD^+^ import versus intramitochondrial synthesis to mitochondrial NAD homeostasis and cellular physiology remains under investigation. While SLC25A1 has also been proposed to play a role in these processes, its precise contribution to mitochondrial bioenergetics remains a subject of ongoing debate and requires more stringent validation [14,75,76]. In contrast, NADH itself cannot cross the inner mitochondrial membrane. Therefore, the functional coupling between cytosolic and mitochondrial redox states is mediated by redox shuttle systems, primarily the Malate–Aspartate Shuttle (MAS) and the Glycerol-3-Phosphate (G3P) shuttle, which transfer reducing equivalents rather than NAD(H) molecules across compartments. These shuttles regenerate cytosolic NAD^+^ to sustain glycolysis while simultaneously influencing the NAD^+^/NADH ratios and redox balance of both cytosolic and mitochondrial compartments, thereby affecting ATP production and cellular metabolism [77,78]. Despite the inherent technical difficulties in quantifying these localized pools, the mitochondrial NAD^+^/NADH ratio is typically maintained at a lower level compared to the cytosol, ranging from 7 to 8 [79], reflecting the electron transport chain activity, which rapidly re-oxidizes NADH to NAD^+^. Interestingly, mitochondrial NAD remains stable even when cytosolic NAD^+^ levels are severely reduced, suggesting that mitochondria maintain a dedicated NAD reservoir to preserve their function under cellular stress conditions [33]. Moreover, mitochondrial NAD^+^ can compensate for depletion in other cellular compartments, highlighting the role of mitochondria as central regulators of inter-organelle NAD balance [33].

Mitochondrial NAD metabolism is dynamically regulated by the segregation of mitochondrial subpopulations with distinct metabolic functions. When ATP demand increases, pyrroline-5-carboxylate synthase (P5CS), the rate-limiting enzyme for the reductive biosynthesis of proline and ornithine, becomes sequestered in a subset of mitochondria lacking cristae and ATP synthase. These “biosynthetic” mitochondria have high reductive potential, sustaining lower NAD(P)+/NADP(H) ratios, while the complementary “energetic” subpopulation, enriched in ATP synthase and organized cristae, operates with higher NAD^+^/NADH ratios to maximize oxidative phosphorylation. This segregation is reversible, depends on mitochondrial fusion and fission dynamics and indicates that NAD redox balance within mitochondria changes according to bioenergetic versus biosynthetic needs [80].

#### 2.1.3. Nucleus

The nuclear NAD^+^ pool is critical for processes such as DNA repair, gene expression, and epigenetic modifications mediated by NAD^+^-dependent enzymes such as sirtuins and PARPs [5,23]. The free nuclear NAD^+^ concentration is estimated to be comparable to that of the cytosol, typically around 10–100 µM in COS7 cells and ~100 µM in HEK293T cells [31,71]. Due to the presence of nuclear pore complexes, the free cytosolic and nuclear NAD compartments are generally considered to be in equilibrium, allowing changes in cytosolic redox state to be reflected in the nucleus [71,81].

#### 2.1.4. Endoplasmic Reticulum (ER)

Emerging evidence indicates distinct ER NAD^+^ pools, separate from the cytosolic one and playing roles in regulating ER function and redox homeostasis [82]. The ER lumen maintains a highly oxidizing environment essential for proper protein folding, and while NADPH is generally considered the primary reductant in the ER, NAD^+^-dependent processes, such as ADP-ribosylation, also occur within this compartment [69,82]. The concentrations of NAD^+^ and NADH within the ER have been less extensively quantified compared to mitochondria and the cytosol.

#### 2.1.5. Golgi

NAD^+^ may also play a critical role in the maintenance of Golgi apparatus structure and function. For instance, NAD^+^ is required for the disassembly of the Golgi complex by certain inhibitors—such as Brefeldin A— suggesting a role in ADP-ribosylation events within the Golgi [83]. Also, one isoform of the NAD^+^ synthesizing enzyme, NMNAT2, specifically localizes to the Golgi apparatus, indicating active NAD^+^ metabolism there [84]. Although the exact NAD concentration and NAD^+^/NADH ratio within the Golgi apparatus can be less clearly pinpointed than in the cytosol or mitochondria, it is generally hypothesized that the Golgi—being part of the endomembrane system and closely connected to both the ER and cytosol—maintains a relatively high NAD^+^/NADH ratio, comparable to that of the cytosol. This redox environment would support oxidative modifications and protein-folding processes occurring in this compartment. However, to date, no direct experimental evidence has been reported to substantiate this assumption.

#### 2.1.6. Peroxisomes

Peroxisomes are involved in various lipid metabolism pathways, including fatty acid β-oxidation, and contain NAD-dependent enzymes [69]. Maintenance of NAD homeostasis within peroxisomes seems vital for their function: studies have shown that the peroxisomal NAD^+^/NADH ratio is closely connected to the cytosolic ratio, with specific redox shuttle systems (e.g., malate/oxaloacetate shuttle and glycerol-3-phosphate shuttle, also pyruvate/lactate [69]) regulating this crosstalk and ensuring the availability of NAD^+^ for peroxisomal metabolic reactions [69,85].

#### 2.1.7. Extracellular Space

NAD^+^ is also present in the extracellular space [86]. Probably, the primary source of extracellular NAD^+^ is the release from dying or damaged cells with compromised cell membrane integrity. However, extracellular NAD^+^ may also be regulated by pore-forming proteins [86,87,88]. Extracellular NAD^+^ levels are much lower than intracellular levels, and likely, extracellular NAD^+^ is metabolized by ectoenzymes with roles in cellular signaling. For instance, CD38 and CD157 (NAD^+^ glycohydrolases) break down NAD^+^ into the signaling molecule cyclic ADP-ribose [89,90,91]. Therefore, extracellular NAD^+^ could represent a targetable pool for modulating signaling pathways, with potential implications in inflammation, immunity, and tissue regeneration, if better understood.

### 2.2. Tissue-Specific Concentration

**Figure 4:**
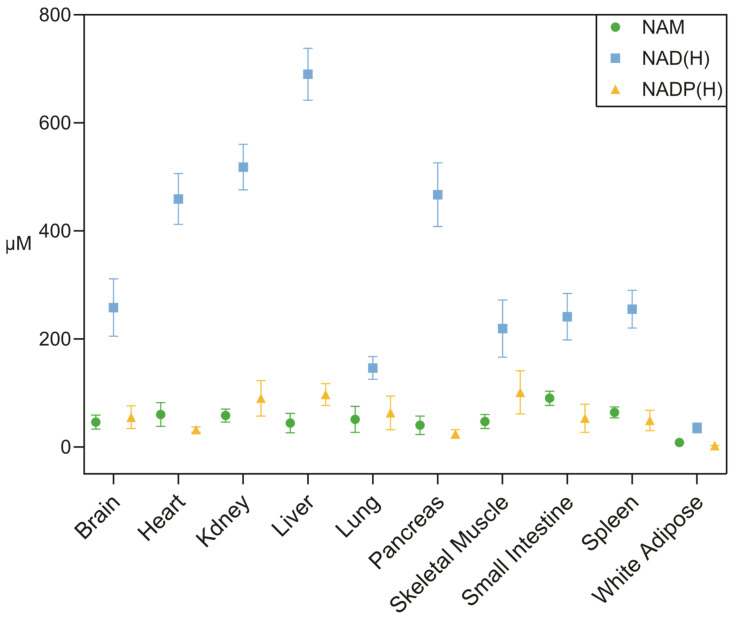
Concentrations of NAM, NAD(H), and NADP(H) in murine tissues. Data are expressed as mean ± s.d., *n* = 4, unit: µM. Data from Liu et al. 2018 [41].

Recently, quantitative metabolomic profiling revealed a pronounced inter-tissue heterogeneity in absolute NAD-related metabolite concentrations in a mouse model, with values ranging from ~690 μM in liver to ~35 μM in adipose tissue (Figure 4) [41]. This gradient suggests that the availability of NAD(H), as well as its related metabolites NAM and NADP(H), depends on tissue-specific biosynthetic capacity, turnover kinetics, and the activity of NAD-consuming enzymes. These differences represent a major challenge for systemic NAD-boosting strategies, since pharmacological or nutritional interventions can have very different effects depending on the metabolic context of each organ [92].

In addition to NAD(H), its precursor nicotinamide (NAM) and the phosphorylated redox pool NADP(H) also display tissue-specific variability (Figure 4). NAM concentrations are generally lower and more homogeneous across tissues, consistent with its role as a circulating intermediate in NAD^+^ turnover and salvage pathways. In contrast, NADP(H) levels exhibit a distinct distribution, with relatively higher concentrations in metabolically active tissues such as liver and kidney, reflecting its central role in anabolic reactions and redox homeostasis [41].

This variability is largely attributed to differential expression patterns of enzymes involved in NAD^+^ biosynthesis and recycling pathways [93]. Notably, tissues with high levels of proliferation or immune activity, such as the small intestine and the spleen, exhibit rapid rates of NAD^+^ turnover, characterized by short half-lives [41]. In contrast, metabolically active but less proliferative tissues, such as skeletal muscle, exhibit significantly slower NAD^+^ turnover. This highlights that NAD metabolism is highly regulated and adapted to the specific needs of each tissue.

Circulating NAD^+^ levels in blood and blood cells have been reported to be substantially lower than those observed in solid tissues, further supporting the existence of pronounced inter-compartment variability [94]. However, direct quantitative comparison across studies remains challenging due to methodological heterogeneity.

Therefore, successful modulation of NAD metabolism must consider not only subcellular compartmentalization but also inter-organ variability in NAD pool size and turnover, as both factors determine the overall physiological impact of NAD-targeted interventions. In addition, potentially tissue-specific import kinetics of NAD or NAD^+^ precursors must be considered.

**Table 3:** Reported concentration of NAD^+^, NADH, and [NAD^+^]/[NADH] ratios in different mammalian cell compartments.

	[NAD^+^]	[NADH]	[NAD^+^]/[NADH] Ratio
NUCLEUS	10–100 µM (free) in COS7 cells (Cercopithecus aethiops Renal cells) [71] ~100 µM (free) in HEK293T (Clonal human embryonic kidney) cells [31] ~260 µM (free) in A375 human melanoma cells [72]	0.1–1 µM (free) in mammalian cells [42]	~700:1 (free estimate) in rat liver cells [95]
CYTOSOL	10–100 µM (free) in COS7 cells (Cercopithecus aethiops Renal cells) [71] ~100 µM in HEK293T (Clonal human embryonic kidney) cells [31]	0.1–1 µM (free) in mammalian cells [42]	~700:1 (free) in rat liver cells 700–2500:1 (free) in human cancer cells [95]
MITOCHONDRIA	~230 µM (free) in HEK293T (Clonal human embryonic kidney) cells [31]	~30 µM (free) in Clonal human embryonic kidney (HEK293T) cells [64]	7–8:1 (free) in rat liver cells [96]
ENDOPLASMIC RETICULUM (ER)	n.d.	n.d.	n.d.
GOLGI	n.d.	n.d.	n.d.
PEROXISOMES	n.d.	n.d.	~700:1 (free) in HEK293 and HeLa [5,69]
EXTRACELLULAR SPACE	n.d.	n.d.	n.d.
WHOLE CELL	[NAD^+^]: 0.2–0.5 mM (Total) in mammalian cells [5] TOT [NAD^+^] + [NADH]: 1–3 mM (free and bound) [95]. Depending on the methods used, species, cell type, and metabolic state.		2–10:1 (Total) depending on species, cell type, and metabolic state [95]

Reported NAD concentrations vary substantially depending on the measurement method, sample preparation, and whether total or free NAD pools are assessed. Therefore, values should be interpreted within the methodological context of each study.

## 3. Therapeutic Strategies Targeting NAD Metabolism

The pivotal role of NAD^+^ in cellular energy metabolism, DNA repair, and signaling pathways has made NAD^+^ metabolism an appealing target for therapeutic interventions across diverse conditions, including age-related diseases, metabolic disorders, and cancers. A broad spectrum of strategies has been developed to either increase NAD^+^ availability or modulate the activity of enzymes involved in its synthesis and consumption. Importantly, the efficacy of these approaches is increasingly recognized to depend on tissue-specific and subcellular NAD dynamics, which may differentially influence therapeutic outcomes.

### 3.1. NAD Precursors and Supplements

One of the most widely explored strategies to enhance intracellular NAD^+^ levels is the administration of NAD^+^ precursors. Nicotinamide riboside (NR) and nicotinamide mononucleotide (NMN) are particularly prominent in this regard, both serving as direct precursors that can be converted to NAD^+^ through salvage pathways [10] (Figure 1). Preclinical studies have consistently shown that NR and NMN oral supplementation increases NAD^+^ levels in multiple tissues, leading to beneficial effects on mitochondrial function, metabolic health, and even lifespan [15].

Clinical studies investigating the effects of NR and NMN supplementation are ongoing and promising. For instance, studies have demonstrated that NR supplementation can safely increase whole blood NAD^+^ levels in healthy adults [97]. Furthermore, clinical trials investigated the effects of NR and NMN on metabolic parameters, muscle function, and cardiovascular health, with some studies reporting improvements in insulin sensitivity, endurance, and inflammatory markers, particularly in older adults or those with pre-existing metabolic diseases [98,99]. However, despite encouraging results, long-term efficacy, optimal dosing strategies, and tissue-specific responses remain incompletely understood. Despite the promising effects of NAD^+^ precursors, their efficacy may be limited by bioavailability, tissue-specific uptake, and metabolic conversion [41]. In this context, alternative strategies have been developed to directly enhance endogenous NAD^+^ biosynthesis.

Among these, small-molecule activators of nicotinamide phosphoribosyltransferase (NAMPT), such as SBI-797812, act by stimulating the rate-limiting step of the NAD^+^ salvage pathway, thereby increasing intracellular NAD^+^ production [100]. In contrast to precursor-based approaches, which depend on uptake and enzymatic conversion, NAMPT activators may provide a more direct and potentially more efficient means of boosting NAD^+^ levels.

Similarly, neuroprotective compounds such as P7C3 have been shown to elevate intracellular NAD^+^ levels, likely through stabilization of NAMPT activity, thereby supporting neuronal survival under conditions of metabolic and oxidative stress. Preclinical studies indicate that P7C3 can improve mitochondrial function and confer protection in models of neurodegeneration, highlighting its potential relevance in age-related neurological disorders [101,102].

However, despite these promising findings, several limitations remain. The long-term safety, tissue specificity, and potential off-target effects of these compounds are not yet fully understood. Moreover, excessive or non-specific activation of NAD^+^ biosynthesis may have context-dependent consequences, potentially supporting pathological processes such as tumor metabolism or maladaptive stress responses. Therefore, further investigation is required to determine their translational potential and to assess whether selective modulation of NAD^+^ synthesis can be achieved in a compartment-specific manner [103,104].

The route of administration appears to play a crucial role in determining the bioavailability and pharmacokinetics of NR and NMN. While NR and NMN can be administered systemically, their metabolic fate is highly context-dependent. Following oral administration, a substantial fraction of NR or NMN is converted to nicotinamide (NAM) in the liver before reaching peripheral tissues, suggesting that systemic NAD^+^ increases often rely on secondary salvage pathways rather than direct precursor uptake. These findings suggest that the route of administration significantly influences the utilization of these precursors in the body [41,105].

While the liver plays a central role in systemic NAD metabolism through de novo synthesis from tryptophan, many cell types possess the enzymatic machinery required for NAD^+^ synthesis, albeit at different capacities [5]. This raises an important distinction between cell-autonomous NAD^+^ production and systemic NAD^+^ supply. Some tissues rely heavily on circulating precursors (e.g., NAM), whereas others maintain local NAD^+^ pools through intrinsic biosynthetic pathways. This balance has significant implications for therapeutic strategies, as NAD^+^ boosting interventions may differentially affect tissues depending on their metabolic autonomy [103,106].

Moreover, the gut microbiome also has a significant impact on NAD^+^ metabolism by converting precursors such as NR, NMN, and NAM into deamidated metabolites through bacterial enzymes like PncA and PncC [107]. Therefore, only a small proportion of NAD^+^ precursors reach tissues unmodified [107].

### 3.2. Enzyme Modulators

Beyond direct NAD^+^ precursor supplementation, modulating the activity of enzymes involved in NAD^+^ synthesis or consumption represents another promising therapeutic approach. Inhibitors of NAD^+^-consuming enzymes, such as CD38 and PARPs, aim to preserve intracellular NAD^+^ levels by limiting its degradation. CD38 is a major NADase responsible for significant NAD^+^ degradation, and its inhibition has shown potential in increasing NAD^+^ levels and improving metabolic function in preclinical models, particularly in the context of aging [108]. Similarly, PARPs are NAD^+^-dependent enzymes essential for DNA repair; however, excessive PARP activation -such as during extensive DNA damage- can lead to NAD^+^ depletion. PARP inhibitors, widely used in oncology, may also indirectly preserve NAD^+^ pools by reducing NAD^+^ consumption, although their systemic metabolic effects remain to be fully characterized [109,110].

Importantly, modulation of NAD metabolism may interact with other pharmacological treatments. Many commonly used drugs, including antipsychotics [111] and chemotherapeutic agents [112,113], can impair mitochondrial function and cellular bioenergetics, thereby influencing NAD^+^ turnover and redox balance. As a result, NAD^+^-boosting strategies may have context-dependent effects, potentially enhancing or counteracting the metabolic impact of concomitant therapies [104].

Alternatively, activating NAD^+^-dependent enzymes, such as sirtuins, has emerged as another strategy. Sirtuins are a family of deacetylases that require NAD^+^ for their activity and play crucial roles in regulating metabolism, inflammation, and aging [114]. Sirtuin activators (e.g., resveratrol analogs for SIRT1) aim to enhance sirtuin activity, thereby mimicking the beneficial effects of caloric restriction [115]. These enzyme modulators provide a more targeted and mechanistically specific approach to manipulating NAD^+^ metabolism, with potential applications in inflammatory conditions, neurodegenerative disorders, and metabolic diseases.

### 3.3. Gene Therapy and NAD Regulation

Genetic strategies manipulate NAD^+^ metabolism by directly influencing the expression of key enzymes involved in NAD^+^ synthesis.

Overexpression of rate-limiting enzymes in NAD^+^ biosynthesis pathways, such as NAMPT or enzymes in the de novo synthesis pathway, could provide sustained increases in cellular NAD^+^ levels [9]. In preclinical studies employing viral vectors to deliver genes encoding for NAD^+^ synthetic enzymes, NAD^+^ levels were successfully increased, and disease phenotypes in models of metabolic dysfunction and neurodegeneration were ameliorated [116]. While still in early stages of development for applications in humans, these approaches offer the potential for long-term reprogramming of NAD^+^ homeostasis, although challenges related to delivery efficiency, tissue specificity, and safety remain significant.

Importantly, the success of gene- and enzyme-based NAD-targeting strategies is likely to depend on their ability to modulate NAD^+^ levels in a compartment-specific manner. Emerging evidence suggests that restoring NAD^+^ in specific organelles, particularly mitochondria, may be more relevant than globally increasing cellular NAD^+^, highlighting the need for spatially targeted therapeutic approaches [4,27,117].

## 4. Targeting NAD-Related Pathways in Aging and Diseases

NAD^+^ decline represents a central metabolic node linking aging to disease, but its impact is highly dependent on subcellular compartmentalization and tissue-specific bioenergetic demands.

Age-associated NAD^+^ decline contributes to multiple interconnected cellular dysfunctions, including mitochondrial impairment, genomic instability, deregulated nutrient sensing, and chronic inflammation [24,116]. While the underlying mechanisms–primarily diminished NAMPT activity and heightened consumption by CD38 and PARPs–are well-established and serve as a common upstream driver of NAD^+^ imbalance across multiple tissues [108,118].

Emerging evidence highlights alterations in subcellular NAD^+^ distribution as a critical, yet underappreciated, feature of aging [23,27].

In aged cells and tissues, cytosolic and nuclear NAD^+^ pools often deplete preferentially, whereas mitochondrial NAD^+^ is relatively preserved through buffering mechanisms, prioritizing oxidative phosphorylation under stress [23,44].

These alterations are tightly linked to widespread changes in cellular bioenergetics, including impaired glycolytic flux (due to reduced GAPDH activity), decreased tricarboxylic acid (TCA) cycle turnover, altered lipid metabolism (including impaired β-oxidation), and compromised mitochondrial respiration, all of which critically depend on NAD^+^ availability as a central redox cofactor and regulator of metabolic fluxes [17,119].

However, prolonged aging eventually erodes this reserve, exacerbating redox imbalance and energetic failure.

In neurodegenerative disorders such as Alzheimer’s disease (AD) and Parkinson’s disease (PD), reduced NAD^+^ availability disrupts neuronal bioenergetics at multiple levels, including impaired glycolysis, mitochondrial dysfunction, and defective proteostasis. These alterations converge on reduced ATP production and increased oxidative stress, ultimately enhancing neuronal vulnerability [119,120]. Preclinical studies have shown that NAD+ precursors (NR and NMN) can restore glycolytic flux, TCA cycle activity, and lipid metabolism, thereby improving mitochondrial function, reducing pathology, and ameliorating cognitive deficits in AD and PD models [119,120]. Emerging clinical evidence supports beneficial effects on mitochondrial function and oxidative stress markers, although larger trials focused on cerebral bioenergetics are still needed [17,99].

In the aging brain, NAD^+^ depletion reduces the activity of NAD^+^-dependent enzymes, including sirtuins and PARPs, leading to impaired DNA repair and progressive mitochondrial dysfunction. This metabolic failure is both a cause and a consequence of cellular senescence, a state of irreversible cell-cycle arrest that accumulates in the aging CNS. Senescent cells develop a Senescence-Associated Secretory Phenotype (SASP), releasing pro-inflammatory mediators that further disrupt tissue homeostasis. Importantly, upregulation of the NAD^+^-consuming enzyme CD38 in senescent cells acts as a metabolic “sink,” further depleting NAD^+^ pools and amplifying bioenergetic dysfunction and inflammation.

Alterations in the NAD^+^/NADH ratio further constrain oxidative phosphorylation and shift neuronal metabolism toward less efficient energy-producing pathways [17].

Comparable NAD^+^-dependent metabolic alterations are observed in obesity, type 2 diabetes, and cardiovascular diseases [15,121,122]. In these conditions, reduced NAD^+^ availability contributes to insulin resistance, impaired mitochondrial fatty acid oxidation, altered glucose metabolism, and chronic inflammation. Altered NAD^+^/NADH ratios in these diseases reflect systemic redox imbalance and metabolic inflexibility, reinforcing the central role of NAD^+^ in coordinating energy metabolism across tissues.

Preclinical studies in aged animal models have consistently shown that restoring NAD^+^ levels improves mitochondrial function, enhances metabolic flexibility, and reduces inflammation. However, despite these shared mechanisms, therapeutic responses to NAD^+^ modulation can vary substantially depending on tissue type, disease context, and subcellular NAD distribution [99,123,124]. A critical emerging concept is that global increases in NAD^+^ levels may not uniformly restore cellular function, as distinct subcellular NAD pools are differentially regulated and may respond differently to therapeutic interventions [23].

Importantly, NAD^+^ modulation exhibits context-dependent effects. While increasing NAD^+^ levels can restore metabolic and mitochondrial function, excessive or non-targeted NAD^+^ boosting may also support pathological processes, including tumor metabolism, chronic inflammation, or maladaptive stress responses [125,126]. In addition, NAD-targeting strategies may interact with other pharmacological treatments that influence cellular bioenergetics. For instance, many antipsychotics and psychotropic drugs impair mitochondrial function and disrupt NAD^+^/NADH redox balance. Therefore, the concomitant administration of NAD^+^ boosters could lead to synergistic or antagonistic effects on cell survival and energetic recovery, a factor that must be carefully evaluated in clinical settings for patients with neurodegenerative or psychiatric comorbidities [127].

Overall, the impact of NAD^+^ decline is highly context-dependent and shaped by tissue-specific metabolism and subcellular compartmentalization. As a result, global NAD^+^ boosting strategies may not uniformly restore cellular function, highlighting the need for spatially and temporally controlled therapeutic approaches [27].

Precise quantification of subcellular NAD^+^ dynamics will be instrumental in guiding such targeted therapies and enabling more personalized interventions.

## 5. Challenges and Limitations in NAD Modulation

The preceding sections highlight that NAD metabolism is not only dynamically regulated but also highly compartmentalized across cellular organelles, with distinct pools exhibiting unique concentrations, redox ratios, and responses to stressors. This subcellular organization, together with the tissue-specific regulation of NAD biosynthesis and consumption, poses major challenges to therapeutic modulation. Non-targeted interventions may alter total cellular NAD^+^ levels without effectively restoring the NAD pools most relevant for mitochondrial function, redox regulation, or stress adaptation, resulting in variable efficacy and possible off-target effects [5,14,73,80]. Moreover, emerging evidence suggests that NAD^+^ dynamics are influenced by factors such as age, sex, metabolic status, and disease context, implying that uniform NAD-modulating strategies may yield heterogeneous outcomes across individuals [128,129]. Addressing these limitations will require the integration of compartment-specific quantification methods, including biosensors and organelle-resolved analytical approaches, to guide precision interventions and improve translational efficacy [37,70,104].

Despite compelling preclinical evidence and promising initial clinical results, modulating NAD for therapeutic purposes still faces significant challenges and limitations that require careful consideration and ongoing research to refine strategies and mitigate risks.

### 5.1. Safety Concerns and Side Effects

While NAD^+^ precursors like NR and NMN have generally shown good safety profiles in short-term human trials, the potential risks of long-term NAD supplementation remain largely unknown. The cellular machinery involved in NAD^+^ metabolism is incredibly complex and in constant interaction with biochemical processes and molecular pathways. Sustained or excessive increases in NAD^+^ availability may disrupt this metabolic balance, potentially leading to unintended effects on redox homeostasis, signaling pathways, and cellular adaptation mechanisms [10]. For instance, altering NAD^+^ metabolism may affect the activity of NAD-dependent enzymes such as sirtuins and PARPs, with downstream consequences for mitochondrial function, inflammatory responses, and metabolic regulation [4].

Furthermore, because NAD^+^ supports cellular metabolism broadly, excessive or poorly targeted NAD^+^ boosting could, under certain pathological conditions, facilitate maladaptive responses, including the support of inflammatory or proliferative processes [126,130].

Although preclinical studies have demonstrated clear benefits, chronic NAD^+^ boosting in humans requires rigorous evaluation to rule out the possibility of cumulative toxicities or subtle physiological shifts that might only manifest over prolonged periods [131].

### 5.2. Incomplete Clinical Data

A significant limitation in the field is the incomplete clinical data regarding the effects of NAD^+^ boosting in humans.

Most human studies to date have been relatively small in scale, short in duration, and often conducted in healthy young or middle-aged individuals [104].

There is a critical need for larger, well-controlled, long-term clinical trials involving diverse populations, including elderly individuals and those with specific pre-existing conditions, to fully understand the efficacy and safety of NAD-targeting therapies. Important gaps remain regarding optimal dosing regimens, routes of administration, tissue-specific responses, and the long-term consequences of manipulating NAD metabolism in humans [132,133,134,135]. Furthermore, many studies rely on systemic surrogate markers rather than direct assessments of compartment-specific NAD dynamics or clinically meaningful endpoints, limiting the interpretation of therapeutic benefits.

Without comprehensive clinical evidence, the widespread adoption of NAD-based therapies will remain limited.

### 5.3. Potential for Resistance and Translation Challenges

Translating promising preclinical findings in NAD-targeting therapies into effective human treatments presents several challenges, including the potential for resistance.

Biological systems can adapt to sustained metabolic interventions, and compensatory mechanisms may reduce the long-term effectiveness of NAD-modulating therapies.

For example, adaptive changes in NAD^+^ degradation pathways or altered sensitivity of NAD-dependent enzymes may progressively attenuate therapeutic efficacy [10]. Additionally, pharmacokinetic and pharmacodynamic challenges can hinder translation. Ensuring that NAD^+^ precursors or enzyme modulators reach the appropriate tissues and intracellular compartments at effective concentrations remains a major translational barrier.

The unique metabolic environment of different diseases, such as the tumor microenvironment or the central nervous system, can present significant barriers to effective drug delivery and action, ultimately impacting the success of translating NAD-targeting therapies into widespread clinical use [130,136].

## 6. Conclusions

Taken together, NAD metabolism is highly complex, responding sensitively to slight intrinsic and extrinsic clues while regulating fundamental cellular processes.

To fully exploit the therapeutic potential of NAD-targeting strategies, a deeper understanding of the pharmacokinetics, pharmacodynamics, and compartment-specific regulation of NAD metabolism is essential. One particularly underexplored aspect concerns how NAD-targeting interventions affect intracellular NAD compartmentalization and the activity of NAD-dependent metabolic pathways within specific organelles [27].

As highlighted throughout this review, the biological effects of NAD modulation cannot be fully understood without considering the distinct regulation of subcellular NAD pools and their role in maintaining cellular bioenergetics and redox homeostasis [4,17].

Addressing these knowledge gaps will be critical for the development of precise and personalized NAD-modulating strategies that maximize therapeutic efficacy while minimizing adverse or context-dependent effects.

Luckily, the recent technological progress is enabling increasingly precise investigations of intracellular NAD dynamics at the organelle level.

In this review, we have summarized key methodologies for measuring NAD within specific organelles, thereby providing a framework to guide future methodological integration and refinement.

The complexity of NAD makes it an attractive therapeutic target but also demands a more integrated understanding of how measurement methodologies, subcellular compartmentalization, and disease-specific metabolic contexts influence therapeutic outcomes.

Future progress will depend on the development of delivery systems and intervention strategies that allow precise spatial targeting, careful dose optimization, and controlled modulation of NAD metabolism across different disease settings [104].

## Data Availability

No datasets were generated or analyzed during the current study.

## Abbreviations

The following abbreviations are used in this manuscript:

A375	Human Melanoma Cells
ADP	Adenosine Diphosphate
ADPR	Adenosine Diphosphate Ribose
ATP	Adenosine Triphosphate
cADPR	Cyclic Adenosine Diphosphate Ribose
CD38	NAD Glycohydrolase, Cluster of Differentiation 38
CD157	Cluster of Differentiation 157 (bone marrow stromal antigen-1)
COS7	CV-1 (simian) in Origin with SV40 genes (cell line)
DNA	Deoxyribonucleic Acid
DRP1	Dynamin-Related Protein 1
ER	Endoplasmic Reticulum
FIS1	Mitochondrial Fission Protein 1
G3P	Glycerol-3-Phosphate shuttle
GAPDH	Glyceraldehyde-3-phosphate dehydrogenase
HEK293	Human Embryonic Kidney 293 cells
HEK293T	Human Embryonic Kidney 293 cells expressing SV40 large T antigen
HeLa	Human Cervical Cancer Cell Line
HPLC	High-Performance Liquid Chromatography
LC-MS/MS	Liquid Chromatography Tandem Mass Spectrometry
LOD	Limit of Detection
LOQ	Limit of Quantification
MAS	Malate–Aspartate Shuttle
MFF	Mitochondrial Fission Factor
MFN1	Mitofusin 1
MFN2	Mitofusin 2
MID49	Mitochondrial Dynamics Protein of 49 kDa
MID51	Mitochondrial Dynamics Protein of 51 kDa
MRI	Magnetic Resonance Imaging
NA	Nicotinic Acid
NAD	Nicotinamide Adenine Dinucleotide (both oxidized and reduced)
NAD^+^	Nicotinamide Adenine Dinucleotide (oxidized form)
NADH	Nicotinamide Adenine Dinucleotide (reduced form)
NADP	Nicotinamide Adenine Dinucleotide Phosphate (oxidized form)
NADPH	Nicotinamide Adenine Dinucleotide Phosphate (reduced form)
NAM	Nicotinamide
NAMPT	Nicotinamide Phosphoribosyltransferase
NIR	Near-Infrared
NMN	Nicotinamide Mononucleotide
NMNAT	Nicotinamide Mononucleotide Adenylyltransferase
NMR	Nuclear Magnetic Resonance
NR	Nicotinamide Riboside
OPA1	Optic Atrophy 1 Protein
P5CS	Pyrroline-5-Carboxylate Synthase
PARP	Poly(ADP-Ribose) Polymerase
PET	Photo-induced electron transfer
SARM1	Sterile Alpha and TIR Motif Containing 1
SASP	Senescence-Associated Secretory Phenotype
SIRT1-7	Sirtuins 1 through 7
SLC25A (MCART1)	Solute Carrier Family 25 Member 51 (Mitochondrial Carrier TripleRepeat 1)
SoNar	Sensor of NAD^+^/NADH Ratio
TCA	Tricarboxylic Acid Cycle (Krebs Cycle)
UV	Ultraviolet
